# Arx revisited: involved in the development of GABAergic interneurons

**DOI:** 10.3389/fcell.2025.1563515

**Published:** 2025-03-28

**Authors:** Akio Tsuboi, Seiich Yoshihara

**Affiliations:** ^1^ Department of Molecular Neuropharmacology, Graduate School of Pharmaceutical Sciences, Osaka University, Suita, Japan; ^2^ Laboratory for Molecular Biology of Neural Systems, Medical Research Center, Nara Medical University, Kashihara, Japan

**Keywords:** Arx, transcription factor, olfactory bulb, cerebral cortex, interneuron

## Abstract

The aristaless-related homeobox (Arx) transcription factor, located on the X chromosome, has been implicated in a wide range of neurological disorders, including intellectual disability and epilepsy, as well as diabetes and pancreatic developmental disorders. In the mouse brain, Arx is expressed not only in the olfactory bulb (OB) and cerebral cortex progenitor cells but also in these gamma-aminobutyric acid (GABA)-releasing interneurons. In the initial study, constitutive Arx knockout (KO) mice showed aberrant migration and a reduction in GABAergic interneurons in the neonatal OB. However, constitutive Arx KO mice with perinatal lethality preclude further analysis in adolescent or adult mice. To overcome this, Arx-floxed mice have been crossed with Cre driver mice to generate conditional KO mice with selective Arx deletion in distinct interneuron progenitors. These studies have identified Arx as a key transcriptional regulator involved in the generation, fate determination, and migration of cortical interneurons. This review focuses on the critical role of Arx in the development of progenitor cells and the migration of interneurons in the mouse OB and cerebral cortex, and discusses differences in Arx mutant-based abnormality between mouse mutants and human patients.

## 1 GABAergic interneurons in the olfactory bulb

**FIGURE 1:**
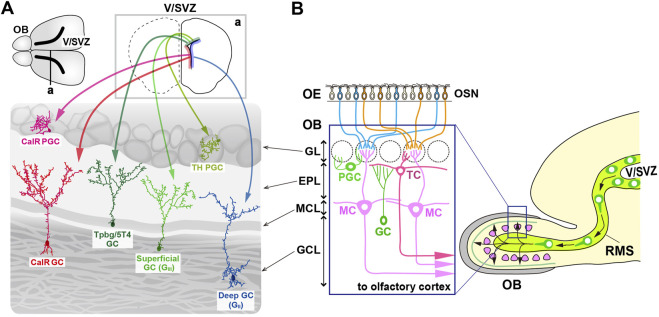
Multiple subtypes of olfactory bulb (OB) interneurons. **(A)** The mammalian OB is structured into distinct layers: the glomerular layer (GL), external plexiform layer (EPL), mitral cell layer (MCL), and granule cell layer (GCL). Olfactory sensory signals from olfactory sensory neurons (OSN) in the olfactory epithelium (OE) are transmitted by excitatory projection neurons such as mitral cells (MCs) and tufted cells (TCs) to inhibitory interneurons like granule cells (GCs) and periglomerular cells (PGCs). **(B)** Distribution of neural stem cells in the ventricular-subventricular zone (V/SVZ) in specific areas. Adult OB interneurons are generated in different subregions of the V/SVZ (upper row; a), migrate through the rostral migratory stream (RMS), and subsequently differentiate into distinct subtypes of mature interneurons in the OB, including PGCs (TH and CalR) and GCs (G_II_, G_III_, Tpbg/5T4, and CalR).

In the olfactory system, odorants are detected by olfactory sensory neurons (OSNs) that express specific odorant receptors in the olfactory epithelium (OE) (Mori and Sakano, 2011; Mori and Sakano, 2021). The axons of OSNs project to distinct glomeruli in the olfactory bulb (OB), where they interact with excitatory projection neurons, promoting the development of dendrites in specific subsets of inhibitory interneurons (Mori and Sakano, 2011; Lepousez et al., 2013; Figueres-Oñate et al., 2014; Mori and Sakano, 2021). OB interneuron progenitors are generated in the ventricular-subventricular zone (V-SVZ) on the lateral ventricle wall, not only during early development but also throughout adulthood (Tong and Alvarez-Buylla, 2014; Figure 1A). These progenitors migrate via the rostral migratory stream (RMS) to the OB, where they differentiate into gamma-aminobutyric acid (GABA)-releasing inhibitory interneurons, including granule cells (GCs) and periglomerular cells (PGCs) (Alvarez-Buylla et al., 2008; Lledo et al., 2008; Whitman and Greer, 2009; Adam and Mizrahi, 2010; Kaneko et al., 2010; Sakamoto et al., 2011; Sequerra, 2014; Figure 1B). In the OB, GCs and PGCs form reciprocal synapses with mitral and tufted cells (M/TCs), receiving glutamatergic inputs from their dendrites and returning GABAergic outputs to their dendrites (Burton, 2017).

GCs are the most abundant non-axonal interneurons in the OB and release GABA from their spiny apical dendrites, which extend into the external plexiform layer (EPL) to interact with the lateral dendrites of M/TCs (Burton, 2017). In contrast, PGCs, which are also non-axonal, have small soma and spatially restricted dendritic branches, and release GABA (and sometimes dopamine) to modulate local glomerular activity (Kosaka and Kosaka, 2011; Galliano et al., 2018). Based on the location of dendritic arborization in the EPL, GCs are further classified into “superficial,” “intermediate,” and “deep” (Mori et al., 1983; Greer, 1987; Takahashi et al., 2018; Figure 1B). Additionally, different subsets of GCs are distinguished by biochemical markers such as calretinin (CalR), Ca2^+^ calmodulin-dependent protein kinase II α (CaMKIIα), oncofetal trophoblast glycoprotein (Tpbg, also known as 5T4), metabotropic glutamate receptor 2 (mGluR2), and neurogranin (Imamura et al., 2006; Batista-Brito et al., 2008; Gribaudo et al., 2009; Merkle et al., 2014; Nagayama et al., 2014; Malvaut et al., 2017). PGCs are further divided into two types: Type 1 expressing tyrosine hydroxylase (TH), the rate-limiting enzyme in dopamine synthesis, and Type 2 expressing calbindin (CalB), CalR, or Tpbg/5T4 (Kosaka et al., 1995; Parrish-Aungst et al., 2007; Toida, 2008; Yoshihara et al., 2012; Nagayama et al., 2014; Figure 1B). CalR and CalB, calcium-binding proteins with EF-hand motifs, maintain calcium homeostasis within neurons and are involved in synaptic plasticity and neurotransmission regulation. Based on the functional properties of CalR and CalB, it might be possible to distinguish the subtypes of GCs and PGCs within the OB.

Embryonic neurogenesis begins around embryonic day (E) 10, when neural epithelial cells in the ventricular zone (VZ) of the lateral ventricle differentiate into radial glial cells (RGCs) (Götz and Huttner, 2005; Turrero García and Harwell, 2017). From E13 to E14, the SVZ is formed via the multiplication of RGCs, and becomes the primary proliferative region. The earliest OB interneurons are generated mainly from the lateral ganglionic eminence (LGE) between E12.5 and E14.5 (Wichterle et al., 1999; Wichterle et al., 2001; Tucker et al., 2006; Kohwi et al., 2007; Batista-Brito et al., 2008). Progenitor cells from the dorsal LGE, expressing transcription factors such as Dlx2, Gsh2 (Gsx2), and Er81 (Etv1), give rise to all major OB interneuron subtypes (Wichterle et al., 2001; Stenman et al., 2003; Qin et al., 2017). Mutations in these and other transcription factors, such as Arx or Sp8, lead to a significant reduction in the number of GABAergic interneurons in both the GC layer (GCL) and glomerular layer (GL) (Stenman et al., 2003; Yun et al., 2003; Yoshihara et al., 2005; Waclaw et al., 2006; Li et al., 2018; Guo et al., 2019).

OB interneuron neurogenesis continues after birth, peaking within the first few weeks of life (Batista-Brito et al., 2008; Figure 1A). Although the rate of neurogenesis declines with age, the ability to generate new neurons persists throughout adulthood in the SVZ, which remains a proliferative region (Alvarez-Buylla and Garcia-Verdugo, 2002; Tramontin et al., 2003; Obernier and Alvarez-Buylla, 2019). Fate mapping studies have shown that the postnatal SVZ contains heterogeneous pools of neural stem cells originating from the medial ganglionic eminence (MGE), LGE, and embryonic cortical regions, which remain quiescent until activated in adulthood (Young et al., 2007; Fuentealba et al., 2015; Furutachi et al., 2015). LGE- and cortical-derived progenitors give rise to distinct populations of OB interneurons, with cortical progenitors predominantly producing CalR-positive interneurons, but with LGE progenitors producing CalB-positive interneurons. Both progenitor pools contribute to the generation of TH-expressing interneurons (Young et al., 2007).

## 2 Aristaless-related homeobox (Arx) transcription factor

**FIGURE 2:**
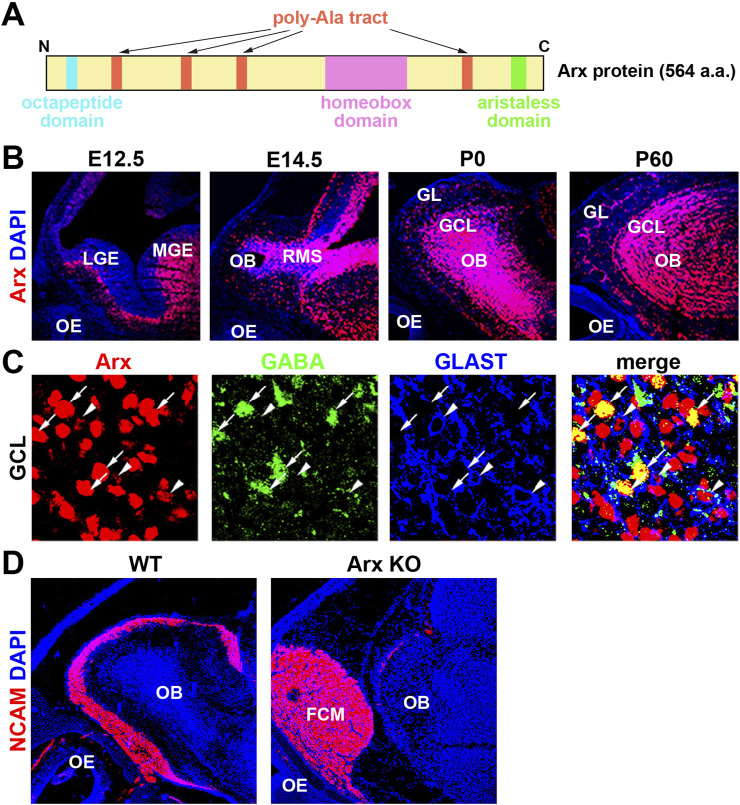
Axons of olfactory sensory neurons (OSNs) fail to enter the OB in Arx KO mice. **(A)** Schematic representation of the homeobox transcription factor Arx. **(B)** Arx is expressed throughout development in the OB but not the OE. Sagittal sections of E (embryonic day) 12.5, E14.5, P (postnatal day) 0, and P60 wild-type mice were labeled with anti-Arx antibody (red) and counterstained with DAPI (4′,6-diamidino-2-phenylindole, blue: nuclei). LGE: lateral ganglionic eminence, MGE: medial ganglionic eminence, RMS: rostral migratory stream. GL: glomerular layer. These figures were taken from Figure 1 of Yoshihara et al. (2005), with permission from the journal. **(C)** Arx is expressed in interneurons (GABA+) and radial glial cells (RGCs, GLAST+) of the OB. Enlarged view of the granule cell layer (GCL), triple-labeled sections of Arx (red), GABA (green: GCs), and GLAST (glutamate transporter, blue: RGCs). Arrows: Arx+ and GABA + GCs. Arrowheads: Arx+ and GLAST + RGCs. These figures were taken from Figure 1 of Yoshihara et al. (2005), with permission from the journal. **(D)** Immunofluorescence labeling of NCAM (neural cell adhesion molecule, red: olfactory axons) and DAPI staining (blue: nucleus) on parasagittal sections of wild-type and Arx-deficient mice at P0. In Arx mutant mice, OSN axons fail to reach the OB and terminate in an axon-tangled structure, termed the fibrocellular mass (FCM). These results suggest that Arx regulates the axonal projection of OSNs through the proper development of either RGCs or interneurons in the OB.

Aristaless-related homeobox (Arx) is a transcription factor containing a paired homeodomain (HD) that is located on the X chromosome. It functions as both an activator and a repressor (Miura et al., 1997; Friocourt and Parnavelas, 2010; Olivetti and Noebels, 2012). In addition to the HD, Arx includes a conserved aristaless domain, an octapeptide domain, and four poly-alanine (Ala) tracts (Friocourt and Parnavelas, 2010; Figure 2A). Mutations in Arx are associated with a broad spectrum of phenotypes, which can be categorized into three primary groups: (1) mutations resulting in truncated proteins, which cause severe intellectual disabilities (ID), autism spectrum disorders (ASD), epilepsy, and brain malformations, particularly the deletion of the corpus callosum (Scheffer et al., 2002; Strømme et al., 2002a; Uyanik et al., 2003), (2) mutations that cause ID, ASD, and epilepsy without structural brain malformations, and (3) missense mutations and in-frame expansions of the first two poly-Ala tracts (Strømme et al., 2002a; Strømme et al., 2002b; Kato et al., 2003). Poly-Ala tract expansion (PAE) mutations have been identified in nine genes, eight of which, including Arx, encode transcription factors (Albrecht and Mundlos, 2005; Messaed and Rouleau, 2009). Unlike polyglutamine repeats, which are more commonly studied, PAEs are typically short (less than 20 Ala residues) and cause developmental defects similar to those seen in Arx, suggesting a shared underlying molecular or genetic mechanism for PAE-related disorders (Albrecht and Mundlos, 2005; Messaed and Rouleau, 2009).

Arx is expressed during development in the nervous system, pancreas, and testes, with its expression continuing in the brain, muscles, heart, and liver in adult mice (Kitamura et al., 2002; Colombo et al., 2004). In the brain, Arx is not only expressed in progenitor cells of the cerebral cortex but also in postnatal GABA-containing interneurons, indicating a potential role in interneuron migration and the development of the cerebral cortex (Colombo et al., 2004; Friocourt et al., 2008; Colasante et al., 2015). Knockout (KO) mouse models, as well as Arx HD mutations, have recapitulated severe epilepsy phenotypes observed in Arx-related disorders.

## 3 Abnormalities in the olfactory system due to Arx deficiency

In the initial study, constitutive Arx KO mice at postnatal day (P) 0 show aberrant migration and a reduction in GABAergic interneurons in the OB (Yoshihara et al., 2005; Figure 2D). Several abnormalities in cell organization, differentiation, and axonal projection were observed in the developing olfactory system of Arx KO mice. OB interneurons, including GCs and PGCs, arise from progenitors in the LGE and migrate rostrally through the RMS to the OB (Luskin, 1998; Wichterle et al., 2001). Arx is strongly expressed in these interneurons and their progenitors, including radial glial cells (RGCs), in the OB and RMS (Figures 2B, C). In Arx KO mice, the proliferation and migration of interneurons to the OB are severely impaired, leading to accumulation of OSN axons at the entrance to the OB. This is similar to the phenotype in the neocortex, to which migration of their interneurons from the MGE is disordered (Kitamura et al., 2002). While the birthplaces, migration routes, and final destinations of interneurons in the cerebral cortex and OB differ, a common mechanism underlying directional neuronal migration likely involves Arx, which may regulate the expression of downstream genes in a cell-autonomous manner. However, the expression patterns of candidate downstream molecules (PSA-NCAM, Robo/Slit, Eph/ephrin, integrin, and Dcc), which may control the migration of OB interneurons, do not differ between Arx KO and wild-type mice (Yoshihara et al., 2005). Additionally, both wild-type and Arx KO mice exhibit a rudimentary RMS glial tube composed of RGCs and astrocytes extending from the SVZ to the OB (Hartfuss et al., 2001; Yoshihara et al., 2005).

In Arx KO mice, the subpopulations of GABAergic interneurons and TH-positive cells were completely absent from the OB (Yoshihara et al., 2005). Furthermore, the expression of Nurr1, a transcription factor crucial for the differentiation of TH-positive OB interneurons, was absent in the mutant mice (Backman et al., 1999; Liu and Baker, 1999). These findings suggest that Arx deficiency disrupts the differentiation of specific interneuron subtypes in the OB. One plausible explanation is that Arx acts upstream of Nurr1 and TH in the differentiation cascade, though it is also possible that progenitors of TH-positive interneurons fail to receive appropriate differentiation signals from the OB due to impaired migration.

Although Arx is not expressed in mitral cells (MCs), abnormalities in the MC layer (MCL) were observed in Arx KO mice, including a thicker and irregular outline of the layer (Yoshihara et al., 2005). Given that an increased number of interneurons from the RMS contributes to OB expansion during late embryonic stages, the disruption of the MCL in Arx KO mice may stem from a reduction in the GCL caused by the failure of interneurons to migrate into the OB. It is also possible that Arx plays a role in the progenitor cells of OB projection neurons, as RGCs serve as progenitors for many brain neurons (Anthony et al., 2004). Abnormal layer formation in OB projection neurons could thus result from cell-autonomous defects in RGCs due to Arx deficiency. Alternatively, the defect could involve a failure of signaling from OB interneurons, RGCs, or OSNs that normally guide the projection pattern of OSNs. In Arx KO mice, most OSN axons fail to reach the OB, terminating instead in a disorganized structure called the fibrocellular mass (FCM), located in front of the OB (Figure 2D).

Several members of the Dlx transcription factor family (Dlx1, Dlx2, Dlx5) play critical roles in the development of the olfactory system. These factors are expressed sequentially, differentially, and in overlapping patterns in OB interneurons and their progenitors (Bulfone et al., 1998; Levi et al., 2003; Long et al., 2003). In Dlx1/Dlx2 double KO mice, severe defects in the proliferation and migration of OB interneuron are observed, with these interneurons being completely absent (Bulfone et al., 1998). In contrast, Dlx5 KO mice exhibit milder phenotypes, which resemble those observed in Arx KO mice, including reduced OB size, impaired migration of OB interneurons, disrupted the MCL, and abnormal axonal projection of OSNs that form the FCM (Levi et al., 2003; Long et al., 2003). It has been reported that Dlx1/2 have key roles in guiding the fate specification and migration of OB interneurons by promoting Arx, Etv1, Pbx3, Prokr2, Sp8, Sp9, and Tshz (Yoshihara et al., 2005; Waclaw et al., 2006; Long et al., 2007; Guo et al., 2019).

In Arx KO mice, the projection pattern of OSNs shows defects in a non-cell autonomous manner: most of the OSN axons fail to reach the OB and terminate in the FCM. The possibility of reciprocal influences between the OE and OB during induction and development has been proposed and widely studied (López-Mascaraque and de Castro, 2002). In rats, the arrival of pioneer OSN axons in the OB regulates cell cycle dynamics and the rate of differentiation of neural progenitor cells, inducing the formation of the OB (Gong and Shipley, 1995). These studies suggest that the OE somehow affects the development of the OB. Is FCM formation due to a deficit originating from the OB rather than the OE? This has been primarily investigated using extratoes (Xt/Xt) mice (St John et al., 2003), which carry a Gli3 mutation. In these mice, the OB is entirely absent, and the sparse OB projection neurons on the rostral surface of the forebrain undergo apoptosis (Hui and Joyner, 1993; St John et al., 2003). In contrast, the OE develops normally in terms of its gross morphology and the expression of signaling molecules, including odorant receptors (Sullivan et al., 1995). However, OSN axons fail to reach the telencephalon and instead terminate in an abnormal structure known as the FCM (St John et al., 2003). These findings suggest that while the OB does not influence cell proliferation or differentiation in the lateral OB, it may play a crucial role in directing OSN axon guidance.

In Arx KO mice, only a small proportion of OSN axons contact the OB, while most fail to reach the OB and terminate in the FCM (Figure 2D). Since Arx is not expressed in OSNs, it has been hypothesized that Arx regulates the expression of one or more guidance signals produced by interneurons and RGCs in the OB to ensure proper OSN axon innervation. To further investigate the molecular mechanisms underlying these observations, microarray was performed to compare gene expression levels between the OBs of wild-type and Arx KO mice. Differential expression analysis revealed alterations (decrease) in genes implicated in neuronal proliferation and migration, such as the cell adhesion molecule Plexin C1 and the cell proliferation regulator Prc1 (polycomb repressive complex 1), including Ring1B that may regulate the differentiation potential of neural stem cells to neurons and glia (Román-Trufero et al., 2009). To determine whether these candidate genes directly regulate interneuron proliferation and migration in the OB, future studies should employ loss- and gain-of-function experiments.

## 4 Abnormalities in the cerebral cortex due to Arx deficiency

Cortical interneurons constitute a diverse population with widely varying morphology, connectivity, and activity patterns (Kepecs and Fishell, 2014). These neurons originate from progenitor cells located in the embryonic proliferative zones known as the MGE, caudal ganglionic eminence (CGE), and LGE (Kepecs and Fishell, 2014). Each ganglionic eminence gives rise to a distinct subset of interneurons; however, the genetic programs governing interneuron fate specification and maintenance remain incompletely understood. The first signs of interneuron diversity appear in the region-specific expression of a limited set of transcription factors within the basal ganglia primordium (Yun et al., 2003; Flames et al., 2007). For instance, the homeobox transcription factor Nkx2.1 is expressed throughout the MGE but is absent in the CGE and LGE (Shimamura et al., 1995). In contrast, the LIM-homeodomain transcription factor Lhx8 is expressed only in specific subdomains of the MGE (Flames et al., 2007). Nevertheless, how these initial heterogeneities contribute to the extensive diversity of adult interneurons remains unclear, further complicated by the fact that many subcortical projection neurons, such as those in the basal ganglia, are also generated from these regions (Zhao et al., 2003; Nóbrega-Pereira et al., 2010).

Arx is a crucial transcription factor in cortical interneuron development, and its mutations are associated with neurodevelopmental disorders such as developmental epilepsies, ID, and ASD in humans (Lim, 2023). For instance, induction of Arx can rescue loss of MGE-derived somatostatin (Sst) and parvalbumin (Pvalb) cortical interneurons in Lhx6 KO mice (Vogt et al., 2014). Nkx2.1, which is critical for the regional specification of the MGE, in turn induces Lhx6 expression to promote Sst and Pvalb interneuron fate in the cortex (Sandberg et al., 2018). Understanding the role of Arx and its associated transcriptional networks is essential for elucidating the underlying mechanisms of these pathologies. Perinatal lethality of constitutive Arx KO mice precludes further analysis in adolescent or adult mice (Kitamura et al., 2002). Several driver mice in which Cre had been inserted so that its expression would mimic that of genes known to shape the emerging identity, function, and positioning of GABAergic cortical interneurons were created (Taniguchi et al., 2011). Then, Arx-floxed mice have been crossed with the Dlx5/6-Cre driver to generate conditional KO (cKO) mice with selective Arx deletion in interneuron progenitors (Marsh et al., 2016). Dlx5/6-Cre cKO male mice (Arx−/Y) show its deficiency in cortical interneuron progenitors, leading to perinatal lethality. However, Dlx5/6-Cre cKO female mice (Arx−/X) show a reduction in the number of interneurons in the cerebral cortex at perinatal and early postnatal stages.

More recently, based on Arx cKO mice with several Cre drivers, Lim et al. (2024) have identified Arx as a key transcriptional regulator involved in the generation, fate determination, and migration of cortical interneurons by modulating gene transcription networks during brain development. For instance, Arx directly or indirectly regulates genes involved in proliferation and the cell cycle (e.g., Bub3, Cspr3), fate specification (e.g., Nkx2.1, Maf, Mef2c), and migration (e.g., Nkx2.1, Lmo1, Cxcr4, Nrg1, ErbB4). First, the loss of Arx in the SVZ of the ganglionic eminences delays cell cycle exit, presumably disrupting the transition from proliferation to differentiation (Lim et al., 2024). This delay is consistent with the aberrant upregulation of Csrp2 (Zhang et al., 2023), a gene known to promote stem cell-like properties, and Bub3 (Silva and Bousbaa, 2022), a cell cycle checkpoint protein frequently overexpressed in tumor cells. As direct transcriptional targets of Arx, the upregulation of these genes in Arx-deficient interneuron progenitors likely sustains a proliferative state and impairs differentiation. Second, a dramatic reduction in Arx-deficient cortical interneurons is observed, particularly within the marginal zone (MZ) stream (Lim et al., 2024). Nkx2.1, a direct target of Arx, is among the most upregulated genes in the MGE cluster. Given that the downregulation of Nkx2.1 is necessary for post-mitotic cortical interneurons to migrate along the cortical migratory stream (Nóbrega-Pereira et al., 2008), defects in interneuron migration in Arx cKO mice may stem, at least in part, from the failure to downregulate Nkx2.1. Third, another direct target of Arx involved in cortical interneuron migration is Lmo1. The expression of Lmo1 is consistently elevated in Arx cKO, constitutive KO, and Arx (GCG)^7^ mutant mice (Lee et al., 2014). Interestingly, ChIP-seq analysis and slice culture electroporation studies indicate that Lmo1 directly represses Cxcr4 expression (Lim et al., 2024). The loss of Cxcr4 in Arx-deficient interneurons, along with the ectopic upregulation of the inductive signal Nrg1/ErbB4 (a direct target of Arx), contributes to the failure of interneurons to enter the cortical MZ. These findings offer novel insights into the role of Arx in cortical interneuron development and its disruption in disease.

## 5 Abnormalities in mice vs. humans due to Arx deficiency

Mutations in Arx, an X-linked gene, are implicated in various neurological disorders, including ID, ASD, and epilepsy in humans (Lim, 2023). While mouse models have demonstrated the critical role of Arx in cortical development and interneuron migration, they do not fully recapitulate the phenotypes observed in human patients. For instance, mice with Arx deletion in cortical projection neuron progenitors exhibit hyperactivity and abnormal behavior but do not develop seizures (Simonet et al., 2015). In contrast, mice with a knock-in Arx poly-Ala expansion (PAE) mutation show a reduction in GABAergic interneurons within the cerebral cortex (Kitamura et al., 2009; Lee et al., 2017) and develop seizures (Price et al., 2009; Mattiske et al., 2016; Loring et al., 2021). Furthermore, epilepsy in many patients with Arx PAE mutations is drug-resistant, underscoring the necessity of developing novel therapeutic strategies. Despite the valuable insights gained from these mouse models, they fail to fully capture the role of Arx in human brain development.

Nieto-Estevez et al. (2024) utilized human neural organoid models derived from male patients with Arx PAE, which harbors eight additional Ala residues in the second poly-Ala tract of Arx. In human cortical organoids that have been generated from induced pluripotent stem cells derived from the patients, Arx PAE causes premature differentiation of RGCs and a depletion of these progenitor cells at the initial stage, followed by a subsequent reduction in GABAergic cortical interneurons at the later stage (Nieto-Estevez et al., 2024). As interneurons originate in the ganglionic eminence and migrate tangentially, the reduction of interneurons in the cortex suggests that Arx affects neuronal migration. Arx PAE promotes the expression of Cxcr4 and accelerates interneuron migration (Beguin et al., 2013); yet, accelerated migration does not lead to increased interneurons in the cortex. It is possible that interneurons with Arx PAE keep moving because they fail to encounter their final target. Defects in GABAergic cortical interneurons contribute to hyperactivity, mirroring the phenotypes observed in Arx mutant mouse models and human patients. Such *in vitro* studies provide valuable insights into the pathological mechanisms underlying Arx PAE mutations and offer a promising human-based platform for developing potential therapeutic interventions.

## Abbreviations

Arx, aristaless-related homeobox; ASD, autism spectrum disorder; cKO, conditional knockout; FCM, fibrocellular mass; GABA, gamma-aminobutyric acid; GC, granule cell; HD, homeodomain; ID, intellectual disability; M/TC, mitral and tufted cell; OB, olfactory bulb; OE, olfactory epithelium; OSN, olfactory sensory neuron; PAE, poly-Ala expansion; PGC, periglomerular cell; RGC, radial glial cell; RMS, rostral migratory stream; TH, tyrosine hydroxylase; V-SVZ, ventricular-subventricular zone.
